# Adhesion of *Toxoplasma gondii* tachyzoite-infected vehicle leukocytes to capillary endothelial cells triggers timely parasite egression

**DOI:** 10.1038/s41598-017-05956-z

**Published:** 2017-07-18

**Authors:** Minami Baba, Tatiana Batanova, Katsuya Kitoh, Yasuhiro Takashima

**Affiliations:** 1 Department of Veterinary Parasitology, Gifu University, 1-1 Yanagido, Gifu, 501-1193 Japan; 2 The United Graduate School of Veterinary Sciences, Gifu University, 1-1 Yanagido, Gifu, 501-1193 Japan; 3 Center for Highly Advanced Integration of Nano and Life Sciences, Gifu University (G-CHAIN), 1-1 Yanagido, Gifu, 501-1193 Japan

## Abstract

Intracellular pathogens have numerous strategies for effective dissemination within the host. Many intracellular pathogens first infect leukocytes, which they use as a vehicle to transport them to target organs. Once at the target organ, intracellular parasite *Toxoplasma gondii* can cross the capillary wall in extracellular form by infecting endothelial cells. However, after egression from leukocytes, extracellular parasites face the risk of host immune attack. In this study, observation of infected mouse organs, using a method that renders tissue transparent, revealed that adhesion of tachyzoite-infected leukocytes to endothelial cells triggers immediate egression of the parasite. This signal enables the parasite to time egression from its vehicle leukocyte to coincide with arrival at a target organ, minimizing the opportunity for immune attack during the transition from a vehicle leukocyte to capillary endothelial cells.

## Introduction

*Toxoplasma gondii* is an obligate intracellular parasite responsible for congenital infections, abortion and opportunistic diseases in immunodeficient individuals. *T*. *gondii* can infect almost all organs of the body including the brain, heart, muscles and lung^1^. Infection by *T*. *gondii* mainly occurs via oral ingestion, and the ingested parasites invade from the small intestine^2, 3^.

It has been reported that *T*. *gondii* tachyzoites infect leukocytes in the lamina propria extravascular space^4, 5^ and that *T*. *gondii*-infected leukocytes in the general circulation transport the tachyzoites to the peripheral organs^6–8^. However, it is still unknown how *T*. *gondii* transit from the infected leukocytes in the general circulation to solid organs. Recently, it has been reported that extracellular *T*. *gondii* tachyzoites in the capillary vessel could infect and replicate in endothelial cells^9^. This study showed that *T*. *gondii* tachyzoites pass through the vascular wall in a motile extracellular form. In the bloodstream, extracellular microorganisms face host immune attack. Although *T*. *gondii* tachyzoites display a certain level of resistance to complement^10^, extracellular tachyzoites directly injected into mouse tail veins rarely arrived at the target organs^7^, indicating that their resistance levels are insufficient for continued survival in the bloodstream. Therefore, for effective transition from leukocytes in the general circulation to solid organs, *T*. *gondii* must egress from the vehicle leukocyte near to the target organ thereby allowing egressed extracellular parasites to immediately enter target cells.

In this study, we revealed that *T*. *gondii* senses the arrival of their vehicle leukocytes at the target organs and then immediately egresses out from the host leukocyte. As an obligate intracellular parasite with limited capacity for extracellular survival, *T*. *gondii* does not randomly egress out from its vehicle cell but rather senses the location within the host body and times egression to maximize its chances of survival.

## Results

### Tachyzoite-infected leukocytes remain in the lungs

**Figure 1:**
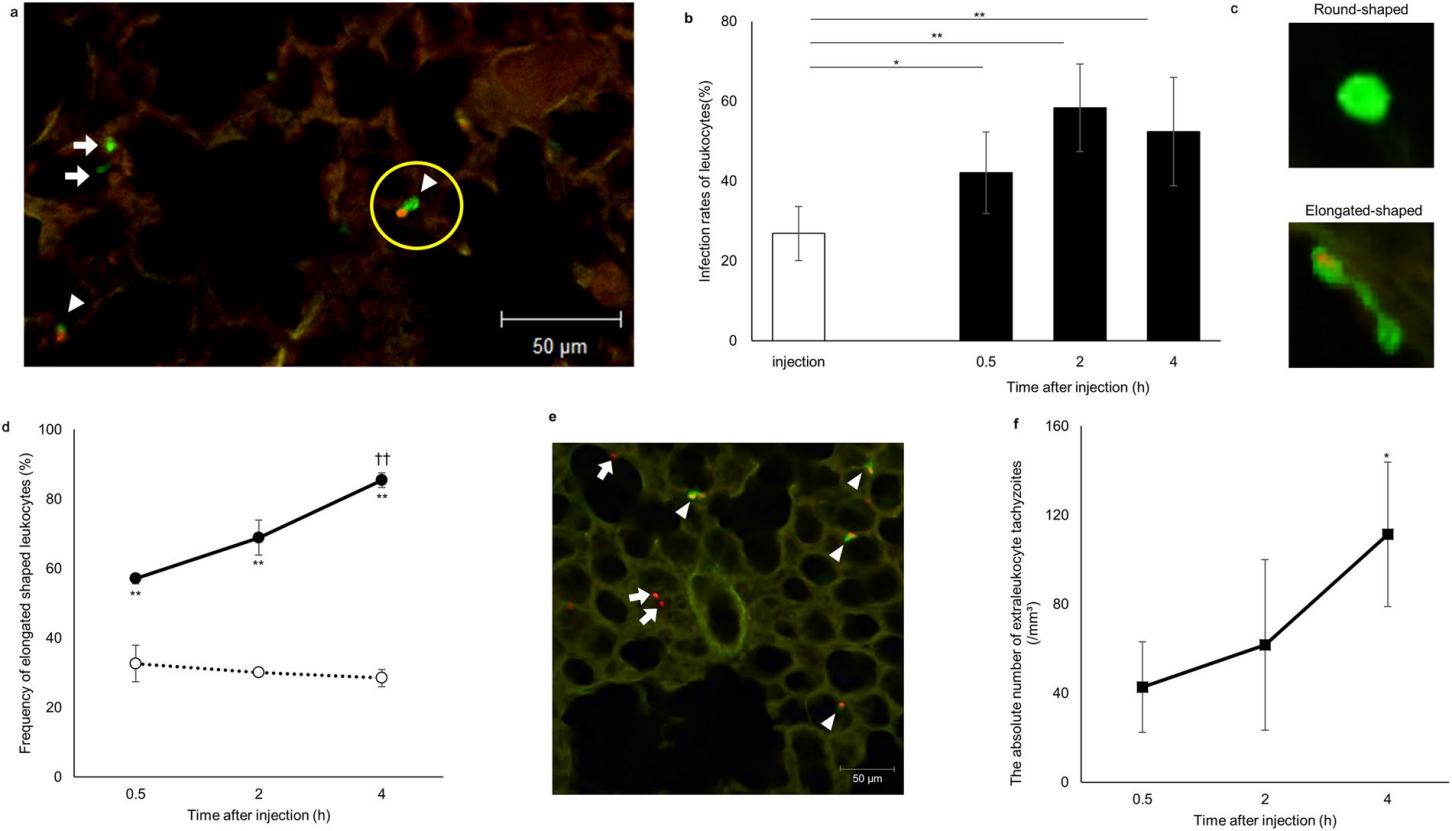
Preferential attachment of infected leukocytes in the lung. (**a**) Representative confocal scanning laser microscope image of the lung at 30 min post injection of leukocytes from a GFP mouse. The circle indicates a red fluorescent tachyzoite-infected leukocyte. Arrows and arrowheads show non-infected and infected leukocytes, respectively. (**b**) Frequency of tachyzoite-infected cells among total GFP-positive leukocytes at the moment of injection (white) and in the lung (black). Results are presented as the mean ± S.E. Data were from three independent experiments and analysed using one-way ANOVA. Scheffe F-test was performed as a post hoc analysis. *p < 0.05 and **p < 0.01. (**c**) Representative images of round shaped (upper) and elongated shaped (lower) leukocytes. (**d**) Frequency of elongated shaped leukocytes in the lung. Solid and dashed lines indicate the frequency of elongated shaped leukocytes among infected and non-infected GFP-positive leukocytes, respectively, in the lung. Results are presented as the mean ± S.E. Data were from three independent experiments and analyzed using two-way ANOVA. Scheffe F-test was performed as a post hoc analysis. **Statistical difference between infected and non-infected leukocytes (*p* < 0.01). ^††^Statistical difference between 4 h and 30 min post injection (*p* < 0.01). (**e**) Representative confocal scanning laser microscope image of the lung at 4 h post injection of tachyzoite-infected leukocytes. Arrows and arrowheads indicate extraleukocytic tachyzoites and infected leukocytes, respectively. (**f**) The number of extraleukocytic tachyzoites in the lung. Results are presented as the mean ± S.E. Data were from three independent experiments and were analysed using two-way ANOVA. Dunnett’s test was performed as a post hoc analysis. *Significant difference from the extraleukocytic tachyzoite number 30 min after leukocyte infection (*p* < 0.05).

The obligate intracellular parasite *T*. *gondii* is thought to disseminate throughout the host by circulation within tachyzoite-infected leukocytes in the blood^7^. However, it remained to be determined how *T*. *gondii* tachyzoites transit from the general circulation to solid tissues. In this study, we injected tachyzoite-infected leukocytes (infection rate: 19.7–40.4%) into the tail vein of mice and visualized the flow of these leukocytes into the solid organs, lung and liver (Fig. 1a,b and Supplementary Fig. 1). Thirty minutes after injection, the infected leukocytes had reached the lung and liver (Fig. 1a and Supplementary Fig. 1). The infection rate of leukocytes that remained in the lung was statistically higher than that of the injected leukocyte suspension (Fig. 1b). Thirty minutes after injection, over half of the infected leukocytes in the lung appeared elongated in shape (Fig. 1c,d,e). The frequency of detection of elongated cells among the total number of infected cells increased with time (Fig. 1d). By contrast, most of the uninfected leukocytes maintained a round shape throughout the observation period (Fig. 1c and d). These results indicated that tachyzoite-infected leukocytes adhere to solid tissue and remain in the lung more effectively than non-infected leukocytes. Tachyzoite-infected leukocytes in the liver also showed a similar tendency (Supplementary Fig. 1).

### Egression of *T. gondii* tachyzoites from leukocytes

**Figure 2:**
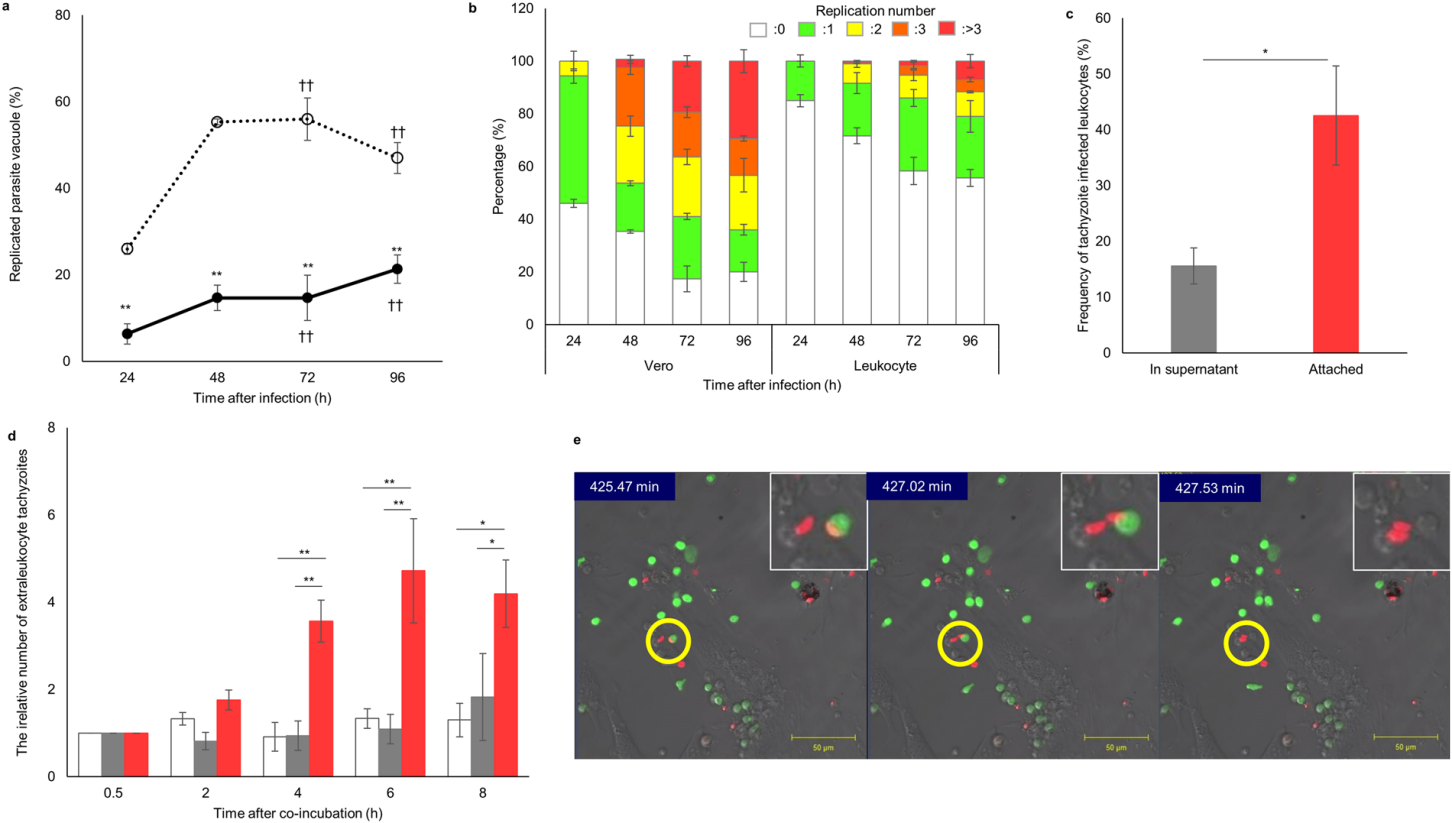
Adhesion to endothelial cells triggers tachyzoite egress from infected leukocytes. (**a**) The percentage of parasite vacuoles including replicated tachyzoites in Vero cells (dotted line) and leukocytes (solid line). Results are presented as the mean ± S.E. Data were from three independent experiments and were analysed using two-way ANOVA. Scheffe F-test was performed as a post hoc analysis. **Statistical difference between leukocytes and Vero cells (*p* < 0.01). ^††^Statistical difference compared with 24 h post infection (*p* < 0.01). (**b**) Replication times of tachyzoites in each vacuole. Results are presented as the mean ± S.E. White: 0, green: 1, yellow: 2, orange: 3, red: ≥4. Data are from three independent experiments. (**c**) Attachment of tachyzoite-infected leukocytes to lung endothelial cells. Tachyzoite-infected leukocytes, with an infection rate of 7.3–25.1%, were co-cultured with lung endothelial cells for 30 min. The frequencies of infected leukocytes among leukocytes in the supernatant (grey bar) and those attached to lung endothelial cells (red bar) are shown. Results are presented as the mean ± S.E. Data were from five independent experiments and were analysed using the Student’s *t*-test (*p < 0.05). (**d**) The relative number of extraleukocytic tachyzoites during *in vitro* culture of tachyzoite-infected leukocytes. The number of extraleukocytic tachyzoites in each culture 0.5 h after infection was considered as “1”. Infected leukocytes were cultured alone (white), cultured with lung endothelial cells separated by 0.4-µm mesh (grey) or attached to lung endothelial cells (red). Results are presented as the mean ± S.E. Data were from five independent experiments and were analysed using the Student’s *t*-test adjusted with the Holm method. Statistical differences were calculated at each time point (*p < 0.05, **p < 0.01). (**e**) Egress of a single tachyzoite from an infected leukocyte attached to lung endothelial cells.

Konradt *et al*. revealed that tachyzoites infect vasculature endothelial cells during the invasion of a solid organ^9^. This study strongly suggested that tachyzoites do not pass through the vascular wall by extravasation of infected vehicle leukocytes but instead in a motile extracellular form. However, it remained to be determined how tachyzoites in blood leukocytes transit to endothelial cells. Based on experiments in a cell culture system, intracellular tachyzoites were thought to receive signal(s) leading to egress after approximately 5 to 7 division cycles^11^. In our study and a previous study, tachyzoite proliferation was shown to be strongly restricted in leukocytes^12^ (Fig. 2a,b). In the present study, to elucidate the timing of tachyzoite egression from leukocytes in the lung, tachyzoite-infected leukocytes were injected into the tail vein and changes in the number of intraleukocytic and extraleukocytic tachyzoites over a time course were evaluated in the lung. Few extraleukocytic tachyzoites remained in the leukocyte suspension at the time of injection (5 × 10^6^ intraleukocytic tachyzoites: 0–1.9 × 10^3^ extraleukocytic tachyzoites/mouse). However, a few extracellular tachyzoites were observed in the lung 30 min after injection and the extracellular tachyzoite number increased during the 4-h observation period (Fig. 1e,f). These results suggested that tachyzoites within leukocytes that remained in the lung rapidly egressed before proliferation. By contrast, a few extracellular tachyzoites were observed in the liver 30 min after injection and the extracellular tachyzoite number remained almost unchanged during the 4-h observation period (Supplementary Fig. 1). We hypothesized that the attachment of tachyzoite-infected leukocytes to endothelial cells triggers egression of tachyzoites. Therefore, we isolated mouse lung endothelial cells and co-cultured them with tachyzoite-infected leukocytes. As observed in the lung (Fig. 1b), tachyzoite-infected leukocytes in the co-culture system also attached to lung endothelial cells more effectively than non-infected leukocytes (Fig. 1e). Extraleukocytic tachyzoites were detected after just 30 min of co-culture, and the number of extraleukocytic tachyzoites increased with time over the entire observation period (Fig. 2d and Supplementary Fig. 2). However, when tachyzoite-infected leukocytes were cultured alone or separated from endothelial cells by a 0.4-µm mesh, the appearance of extraleukocytic tachyzoites was reduced (Fig. 2d and Supplementary Fig. 2). When tachyzoite-infected leukocytes and endothelial cells were co-cultured, following the increase in extraleukocytic tachyzoites, the number of infected leukocytes also decreased with time (Supplementary Fig. 2). These results indicated that tachyzoite egression from leukocytes was required for direct attachment of infected leukocytes to lung endothelial cells. Furthermore, secreted soluble factors from lung endothelial cells did not contribute to parasite egression.

Consistent with these results, we confirmed by time-lapse observations the egression of a single tachyzoite from a leukocyte after the leukocyte attached to an endothelial cell (Fig. 2e and Supplementary Video 1). When the tachyzoite egressed, green fluorescent protein in the leukocyte cytoplasm leaked out, indicating that egression is a cytolytic event.

### Rapid egression of tachyzoites from monocytes

**Figure 3:**
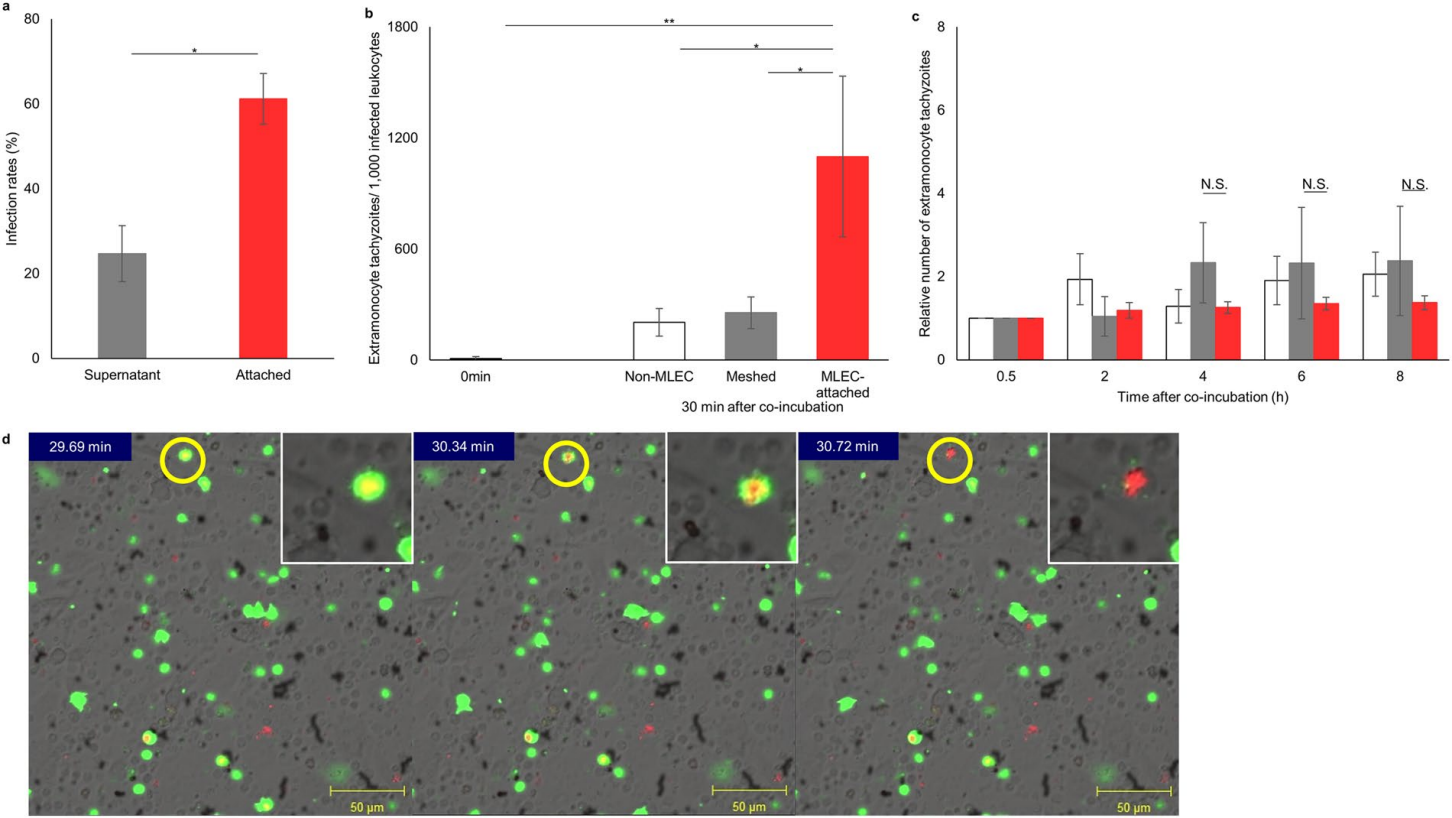
Rapid egress of tachyzoites from monocytes. (**a**) Preferential attachment of tachyzoite-infected monocytes to lung endothelial cells. Tachyzoite-infected monocytes, with an infection rate of 21.1–39.2%, were co-cultured with lung endothelial cells for 30 min. The frequencies of infected leukocytes among the leukocytes in the supernatant (grey bar) and among those attached to lung endothelial cells (red bar) are shown. Results are presented as the mean ± S.E. Data were from three independent experiments and were analysed using the Student’s *t*-test (*p < 0.05). (**b** and **c**) Rapid egress of intramonocytic tachyzoites. (**b**) Comparison between extracellular and infected monocyte numbers. Egressed tachyzoite-infected monocytes were cultured alone (white), co-cultured with lung endothelial cells separated by 0.4-µm mesh (grey) or co-cultured with lung endothelial cells with direct contact (red) for 30 min. Then, the number of extramonocytic tachyzoites in each culture was determined. Results are presented as the mean ± S.E. Data were from three independent experiments and were analysed using one-way ANOVA. Scheffe F-test was performed as a post hoc analysis (*p < 0.05, **p < 0.01). (**c**) The relative number of extramonocytic tachyzoites during *in vitro* culture of tachyzoite-infected monocytes. The conditions were similar to those presented in Fig. 2d. Monocytes were either cultured alone (white), with endothelial cells separated by mesh (grey) or with direct contact to endothelial cells (red). Results are presented as the mean ± S.E. Data were from three independent experiments and were analysed using the Student’s *t*-test at each time point (NS: no statistical difference). (**d**) Egress of tachyzoites from an infected monocyte attached to lung endothelial cells.

Although *T*. *gondii* can infect each type of leukocyte including dendritic cells, monocytes, lymphocytes and neutrophils, myeloid cells are more susceptible to *T*. *gondii* infection than other types of leukocyte and are mainly responsible for transporting tachyzoites to solid organs^6, 13^. Therefore, we repeated several experiments using purified monocytes instead of bulk leukocytes. Similar to bulk leukocytes, tachyzoite-infected monocytes also attached to lung endothelial cells more effectively than non-infected monocytes (Fig. 3a). Thirty minutes after commencing co-culturing of endothelial cells and tachyzoite-infected monocytes, significantly more extramonocytic tachyzoites were observed compared with monocytes cultured alone or with endothelial cells separated by mesh (Fig. 3b,d, Supplementary Fig. 2, and Supplementary Video 2). After that time, further egression was not observed from the monocytes co-cultured with endothelial cells (Fig. 3c and Supplementary Fig. 2). These results indicated that egression of tachyzoites occurred more rapidly (within 30 min) from monocytes than from other types of leukocytes.

### Contribution of CD162, expressed by endothelial cells, to tachyzoite egression

**Figure 4:**
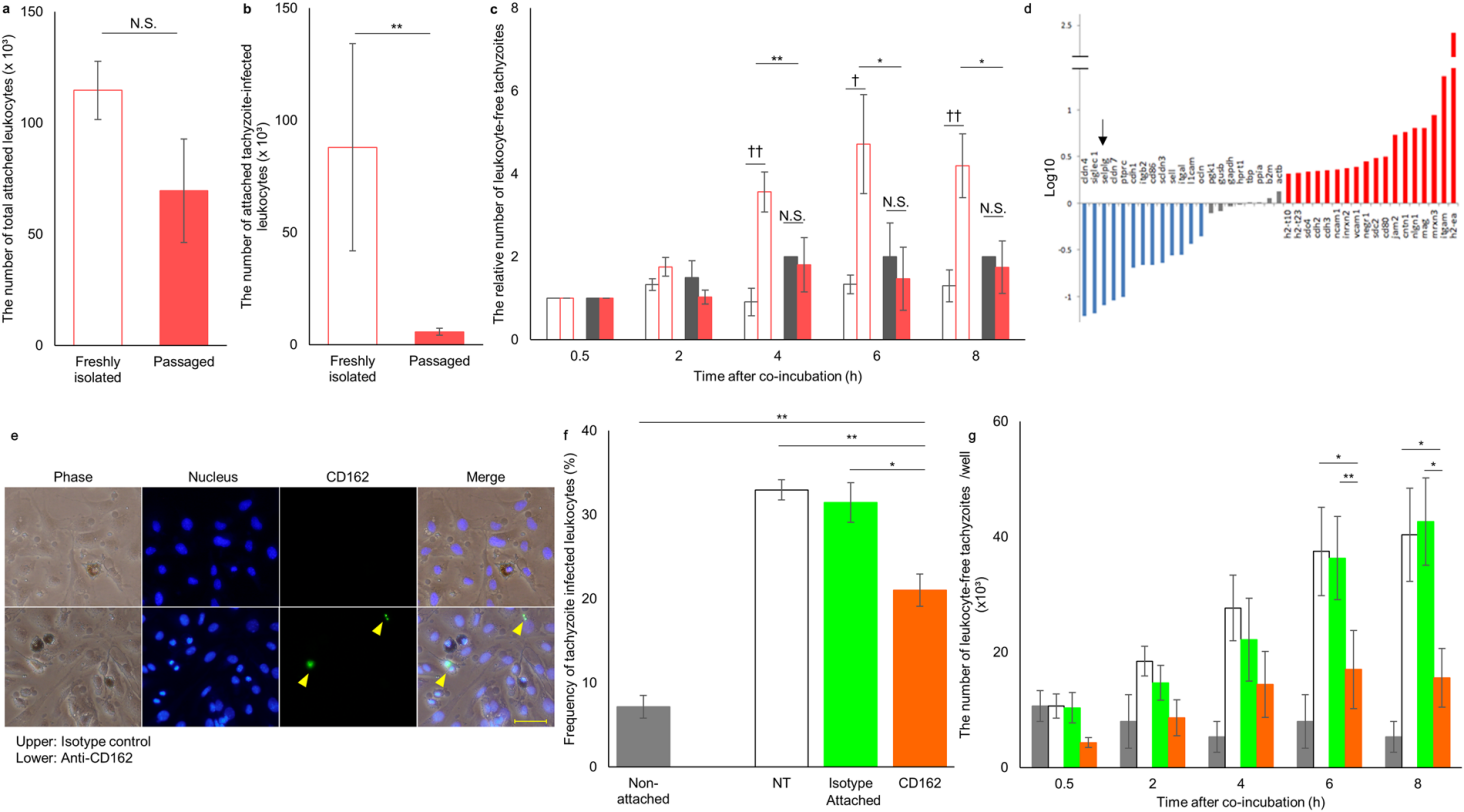
Role of CD162 in tachyzoite egression. (**a** and **b**) The number of leukocytes attached to freshly isolated and 2–3 times passaged endothelial cells (meshed and colored bars, respectively). The numbers of total leukocytes and tachyzoite-infected leukocytes are shown (**a** and **b**, respectively). Results are presented as the mean ± S.E. Data were from three independent experiments. Statistical analysis was carried out using the Student’s *t*-test (**p < 0.01, N.S.: No statistical difference). (**c**) The relative number of extraleukocytic tachyzoites that appeared after co-culture. Meshed and filled bars indicate co-culturing with freshly isolated endothelial cells and 2–3 times passaged endothelial cells, respectively. Leukocytes were cultured either with endothelial cells separated by mesh (grey) or with direct contact to endothelial cells (red). Statistical analysis was carried out using the Student’s *t*-test adjusted with the Holm method. Statistical differences between isolated and passaged endothelial cells (*p < 0.05) and between non-attached and attached leukocytes (^†^ *p* < 0.05, ^††^ *p* < 0.01, N.S.: No statistical difference), respectively, are indicated. (**d**) Relative expression levels of genes encoding adhesion molecules between the freshly isolated and 3-times passaged endothelial cells. Genes for which the expression level was down- or up-regulated by more than 2-fold during serial passage are indicated by blue and red bars, respectively. Grey bars indicate housekeeping genes. Arrows show Selplg (CD162). (**e**) Immunostaining of 0.5% formaldehyde-treated freshly isolated lung endothelial cells. Phase: images with transmitted light, Nucleolus: staining with Hoechst, CD162: staining with isotype control (upper) or anti-CD162 antibody (lower), Merge: the combined image. Arrowhead shows CD162 and the scale bar indicates 50 µm. (**f** and **g**) The effect of blocking CD162 on lung endothelial cells. Lung endothelial cells were pre-treated with anti-CD162 antibody (orange), isotype control antibody (green) or PBS (white). Grey bars indicate the supernatant control. Results are presented as the mean ± S.E. Data were from three independent experiments. Statistical analysis was carried out using the Student’s *t*-test adjusted with the Holm method (*p < 0.05, **p < 0.01). (**f**) Frequency of tachyzoite-infected leukocytes at 0.5 h of co-culturing. (**g**) The number of extraleukocytic tachyzoites that appeared after co-culturing.

Primary mouse vascular endothelial cells maintain their endothelial properties for at least two to three passages, and are used widely as an endothelial cell model during this time period^14, 15^. We also confirmed that there was no statistically significant difference in the total number of attaching leukocytes between freshly isolated and passaged endothelial cells (Fig. 4a). However, when we focused on tachyzoite-infected leukocytes, many more infected leukocytes attached to the freshly isolated endothelial cells (Fig. 4b). In addition, egression of tachyzoites from the infected leukocytes was observed only when the vehicle leukocytes attached to the freshly isolated cells (Fig. 4c and Supplementary Fig. 3). These results indicated that the endothelial cells maintain the majority of endothelial properties but specifically lost the ability to selectively attach to tachyzoite-infected vehicle leukocytes and trigger tachyzoite egress, within only two to three passages. Therefore, we compared the expression level of 88 genes coding for cell adhesion-related proteins between the freshly isolated and three times passaged endothelial cells. After three passages, the expression levels of 13 genes were decreased more than 2-fold (Fig. 4d and Supplementary Table S1). It has been reported that *T*. *gondii*-infected leukocytes attach to vascular endothelial cells via E-selectin, intercellular adhesion molecule-1 (ICAM-1) and vascular cell adhesion molecule-1 (VCAM-1)^16^. However, the expression levels of these three genes were maintained or even increased throughout the three passages (Fig. 4d, Supplementary Fig. 3, and Supplementary Table S1). Among the 13 genes that showed significantly decreased expression levels after three passages was the gene encoding selectin-P ligand (Selplg), also known as CD162, a known mediator of the first step of leukocyte and endothelial cell attachment^17^. This molecule is widely expressed on leukocytes and binds to all types of selectins but predominantly P-selectin. By contrast, only a few reports have shown low level expression of CD162 on the surface of vascular endothelial cells^18, 19^. We carried out immunostaining and confirmed that a portion of freshly isolated lung endothelial cells expressed CD162 even in a non-inflammatory steady state (Fig. 4e and Supplementary Fig. 3). CD162 immunostaining was observed in the nearby nucleus (Fig. 4e). The anti-CD162 antibody also stained unfixed/unpermeabilized cells (Supplementary Fig. 3). This suggested that CD162 is expressed on the surface of freshly isolated lung endothelial cells. The reduction in CD162 expression at the protein level after serial passage of the lung endothelial cells was also confirmed by western blotting analysis and flow cytometry (Supplementary Fig. 3). To examine whether CD162 molecules on the surface of endothelial cells induce tachyzoite egression from leukocytes, the freshly isolated lung endothelial cells were treated with anti-CD162 antibody and excess antibody was removed by washing before co-incubation with tachyzoite-infected leukocytes. Antibody treatment of endothelial cells did not affect the total number of attached leukocytes (Supplementary Fig. 3). When we focused on tachyzoite-infected leukocytes only, it was found that the number of attached tachyzoites-infected cells was statistically slightly decreased by antibody treatment (Fig. 4f). The anti-CD162 antibody had little or no effect on leukocyte adhesion. By contrast, anti-CD162 antibody treatment of the freshly isolated endothelial cells drastically decreased the number of egressed tachyzoites (Fig. 4g). This indicated that CD162 molecules on the surface of the lung endothelial cells contribute to tachyzoite egress from the attaching leukocytes. Consistent with this, treatment with anti-CD162 antibody abolished the time-dependent decrease in the number of tachyzoite-infected leukocytes (Supplementary Fig. 3).

## Discussion

In this study, we demonstrated that tachyzoite-infected leukocytes in the general circulation remain in the lung by effective adhesion to lung endothelial cells and that this adhesion triggers tachyzoite egression from the vehicle leukocyte. Secreted soluble signals from lung endothelial cells did not contribute to parasite egression. However, CD162 molecules expressed on the endothelial cells were found to contribute to egression.

A variety of intracellular pathogenic microorganisms are delivered by infected leukocytes to peripheral organs^20–22^. It had been thought that extravasation of these infected leukocytes transfers the microorganisms from the blood to solid organs beyond the blood vessel wall, in a “Trojan horse”-like manner^23–25^. However, several intracellular microorganisms are reported to invade the brain and placenta^24, 26–28^, where migration of leukocytes out of the blood vessels is barely detectable^29, 30^. Furthermore, it was recently reported that *T*. *gondii* tachyzoites infect endothelial cells of the vasculature and then proliferate before invading solid organs^9^. These results suggested an alternative method by which intracellular microorganisms may pass through the blood vessel wall. Our study revealed insight into how the intracellular microorganism *T*. *gondii* traverses the blood vessel wall. *T*. *gondii* tachyzoites sense the arrival of its hijacked leukocyte vehicle to a target solid organ and time egression accordingly. This strategy effectively minimizes the time lag between egression from vehicle leukocytes and invasion into solid organ target cells, thereby reducing the exposure time to complement and antibodies in the serum.

Increasing of calcium concentration in the parasite cytosol has been recognized as a trigger for *T*. *gondii* tachyzoite egression^31^. This calcium influx into the tachyzoite cytosol originates, at least partially, from the host cell cytosol^32^. It has been reported that the calcium concentration in the leukocyte cytosol increases immediately after leukocytes adhere to endothelial cells^33, 34^. Calcium ions might be transferred from the cytoplasm of the vehicle leukocytes to the cytoplasm of tachyzoites. When leukocytes adhere to endothelial cells, L-selectin, lymphocyte function-associated antigen-1 (LFA-1) and macrophage-1 antigen (Mac-1) on the surface of the leukocytes bind to ligand molecules on endothelial cells, inducing a calcium influx into the leukocyte cytosol^35, 36^. CD162 is a known ligand of L-selectin. In this study, we revealed that CD162, expressed on the surface of endothelial cells, contributes to tachyzoite egression. Binding of CD162 on endothelial cells to L-selectin on infected leukocytes might trigger calcium transfer between the vehicle leukocyte and the tachyzoite. Although CD162 molecules are mainly expressed on the surface of leukocytes^37^, a subpopulation of mature endothelial cells also express CD162^18, 19^. We also confirmed that a subpopulation of freshly isolated mouse lung endothelial cells express CD162 and that CD162 contributes to tachyzoite egress. However, anti-CD162 antibody treatment of endothelial cells only partially inhibited parasite egress from attached leukocytes. This suggested that CD162 is not the only trigger of egress but that other molecule(s) also contribute to this phenomenon. Although *T*. *gondii* can infect almost all organs, this parasite more frequently infects particular organs *in vivo*, such as the lung, brain and eyes^21, 38, 39^. We also found in this study that tachyzoite-infected vehicle leukocytes adhered to solid liver tissue more effectively than non-infected leukocytes, but in contrast to the lung, rapid tachyzoite egression from the vehicle leukocytes rarely occurred. The heterogeneous distribution of CD162 and/or other trigger(s) on the surface of the capillary wall in each organ might cause preferential infection of particular organs and different egression patterns. To reveal the *in vivo* contribution of such trigger molecules on *T*. *gondii* distribution in the host body, further studies using endothelial cell-specific conditional CD162 knockout mice or other trigger molecule knockout mice are necessary. Although the invasion step has been well studied in many intracellular pathogens, the mechanism of egression is poorly understood. Timely egression from vehicle cells and timely transit to subsequent target cells are vital for effective dissemination of intracellular pathogens in the host. Our findings therefore enhance our understanding of this process and provide valuable insight into pathogen dissemination within the host organism.

## Methods

### Animals, parasites and cells

Green fluorescent protein transgenic mice, C57BL/6-Tg(CAG-EGFP) C14-Y01-FM131 Osb (GFP mouse)^40, 41^, and C57BL/6 mice (Charles River Laboratories Japan Inc., Yokohama, Japan) were used. Experiments were performed in accordance with the Gifu University Animal Care and Use Committee guidelines. The experimental protocols using animals were reviewed and approved by the local ethics committee of Gifu University. Red fluorescent protein (DsRed)-expressing transgenic *T*. *gondii*, PLK/RED, were used in this study^42^. Purified tachyzoites^21^ and mouse leukocytes^43^ were prepared as described previously. Briefly, leukocytes were prepared from the whole spleen following cervical dislocation in mice. Spleens were minced and pressed through a nylon mesh with a pore size of 77 μm. The filtrated cells were suspended in 4.5 ml of hemolytic buffer (0.155 M NH_4_Cl in dH_2_O and 0.5 ml Tris–HCl pH 7.65), incubated for 10 min at room temperature, then washed with PBS to remove erythrocytes. The leukocytes were cultured in RPMI 1640 medium supplemented with 7.5% foetal calf serum plus 20 µg/ml of gentamicin. Monocytes were isolated as previously described^44^ with one modification being the use of Dynabeads® Protein G (Thermo Fisher Scientific, Kanagawa, Japan). After the purification procedure, >98% of the cells were CD11b positive. Mouse lung endothelial cells were isolated from 5–8-day-old C57BL/6 mice as previously described^45^.

Leukocytes from GFP mice were mixed with a PLK/RED tachyzoite suspension at a multiplicity of infection of 1–3 in 1 ml of RPMI 1640 medium supplemented with 7.5% foetal calf serum plus 20 µg/ml of gentamicin and then incubated for 24 h at 37 °C in a 5% CO_2_ incubator. Monocytes from GFP mice or C57BL/6 mice were stained with CellTracker™ Green CMFDA (Thermo Fisher Scientific), then mixed with a tachyzoite suspension at a multiplicity of infection of 1, under the same conditions used for the leukocytes. After incubation, the remaining extracellular tachyzoites were removed as described previously^46^.

### Immunostaining

Mouse lung endothelial cells were fixed with 0.5% formaldehyde for 30 min at room temperature and were then washed three times in PBS. The fixed cells were incubated at 37 °C with a polyclonal antibody to P-selectin glycoprotein ligand (CD162) (Cloud-Clone Corp., Wuhan, China) or a rabbit IgG, polyclonal-isotype control (Abcam Corp., Tokyo, Japan) at a concentration of 10 µg/ml. To specifically stain the cell surface and to avoid unexpected permeabilization by formaldehyde, non-fixed cells were also stained in the same manner. Antibody-bound cells were detected using a 1:50 dilution of goat anti-rabbit IgG H&L (FITC) antibody (Abcam Corp.). The stained cells were incubated for 15 min at room temperature with Hoechst 33342 (DOJINDO LABORATORIES, Kumamoto, Japan) at a concentration of 10 µg/ml. The stained samples were observed using a Biozero fluorescence microscope (BZ-8000; Keyence, Osaka, Japan).

### Observation of tachyzoite-infected leukocytes in the tissue

The tachyzoite-infected leukocytes from the GFP mouse were injected into C57BL/6 mice via the tail vein (5 × 10^7^ infected leukocytes/mouse). Then 0.5, 2 and 4 hours after injection, the mice were euthanized by cervical dislocation and the liver and lung were collected. The collected organs were immediately fixed in 4% paraformaldehyde overnight at 4 °C. The fixed organ samples were made transparent as previously described^47^. Briefly, fixed samples were sliced and soaked in α-thioglycerol added to increasing concentrations (from 20–100%) of fructose solution. After treatment with 100% fructose solution, the samples were soaked in the morphology-preserving agent, SeeDB^47^ solution. The transparent samples were observed and leukocytes and tachyzoites in all fields of vision to a 200-µm depth were counted using confocal laser scanning microscopy (LSM700, Carl Zeiss Japan, Tokyo, Japan). During the observation, leukocytes were measured at their major and minor axes, and if the major axis was more than double the minor axis the cell was considered to be elongated in shape.

### Co-culture of tachyzoite-infected leukocytes/monocytes and lung endothelial cells

1 × 10^6^ tachyzoite-infected leukocytes from the GFP mouse were added to a monolayer of lung endothelial cells and incubated for 30 min at 37 °C in a 5% CO_2_ incubator. After incubation, non-attaching leukocytes were removed by washing and leukocytes attached to the endothelial cells were further cultured. As controls, 1 × 10^6^ tachyzoite-infected leukocytes from the GFP mouse were cultured alone or with an endothelial cell monolayer separated by 0.4-µm mesh.

In addition, 1 × 10^5^ tachyzoite-infected monocytes from GFP mice or C57BL/6 mice, stained with CellTracker™ Green CMFDA, were also cultured as described above with minor modifications. In some cases, non-attached monocytes were removed by washing after 10 or 30 min incubation with the endothelial monolayer.

### Primer array analysis

Total RNA was extracted from the freshly isolated or three times passaged mouse lung endothelial cells using an RNA isolation kit “NucleoSpin RNA” (Macherey-Nagel, Duren, Germany) and was then converted to cDNA according to the manufacturer’s instructions. The relative quantities of cDNA of the genes encoding 88 adhesion molecules and eight housekeeping genes from the freshly isolated and three-times passaged mouse lung endothelial cells were examined in real time using a PrimerArray^®^ of mouse cell adhesion molecules (Takara, Kyoto, Japan). The results were analysed using the Primer Array Analysis Tool Ver. 2.1 (Takara).

### Western blotting

Lung endothelial cells and leukocyte lysates were prepared in 2X SDS sample buffer. The cell lysates were loaded onto 5–20% SDS-PAGE gels and western blotting was conducted using a primary antibody against the P-selectin glycoprotein ligand (CD162) (Cloud-Clone Corp.) at 0.5 µg/ml. As a loading control, glyceraldehyde-3-phosphate dehydrogenase (GAPDH, Cloud-Clone Corp.) was included at a concentration of 0.5 µg/ml. The secondary antibodies used were horseradish peroxidase (HRP)-conjugated goat anti-rabbit IgG antibody (dilution 1:3000, Abcam Corp.) and HRP-conjugated rabbit anti-mouse IgG (dilution 1:3000, Thermo Fisher Scientific), respectively.

### Flow cytometry analysis

Mouse lung endothelial cells were fixed with 0.5% formaldehyde for 30 min at room temperature and were then washed three times in PBS. Permeabilized cells were incubated at room temperature with a polyclonal antibody against the P-selectin glycoprotein ligand (CD162) (Cloud-Clone Corp.) or a rabbit IgG polyclonal-isotype control (Abcam Corp.) at a concentration of 50 µg/ml. Antibody-bound cells were detected using a 1:500 dilution of goat anti-rabbit IgG H&L (FITC) antibody (Abcam Corp.).

### Antibody treatment

Lung endothelial cells were incubated with 10 µg/ml of anti-mouse CD162 rat antibody (BD Biosciences), rat IgG1 isotype control antibody (BD Biosciences) or PBS for 30 min at 37 °C in a 5% CO_2_ incubator. After incubation, the lung endothelial cells were washed with PBS three times and used for further experiments.

### Statistical analysis

Student’s *t*-test was used for comparisons between two groups or several pairs of groups and the Holm method was used to adjust the P value when plural pairs were compared. Analysis of variance (ANOVA) was used to compare groups when there were more than two groups. If the results of ANOVA were significant, a post hoc Scheffe F-test was performed. Statistical significance was set at *p* < 0.05.

## Electronic supplementary material

Supplementary Information

Supplementary Video 1

Supplementary Video 2
