# Supplementary material for: Adhesion of Toxoplasma gondii tachyzoite-infected vehicle leukocytes to capillary endothelial cells triggers timely parasite egression
Source: Sci Rep. 2017 Jul 18;7:5675. doi: 10.1038/s41598-017-05956-z (PMC5515940; doi:10.1038/s41598-017-05956-z)
Supplement: Supplementary file 1 — Supplementary Information [file 41598_2017_5956_MOESM1_ESM.pdf]

Adhesion of *Toxoplasma gondii* tachyzoite-infected vehicle leukocytes to capillary endothelial cells triggers timely parasite egression

Minami Baba[1,2,+], Tatiana Batanova[1,+], Katsuya Kitoh[1,2], Yasuhiro Takashima[1,2,3]*

[1]Department of Veterinary Parasitology, Gifu University, 1-1 Yanagido, Gifu 501-1193, Japan.

[2]The United Graduate School of Veterinary Sciences, Gifu University, 1-1 Yanagido, Gifu 501-1193, Japan.

[3]Center for Highly Advanced INtegration of Nano and Life Sciences, Gifu University (G-CHAIN), 1-1 Yanagido, Gifu 501-1193, Japan.

*Corresponding author: atakashi@gifu-u.ac.jp

[+]These authors contributed equally to this study.

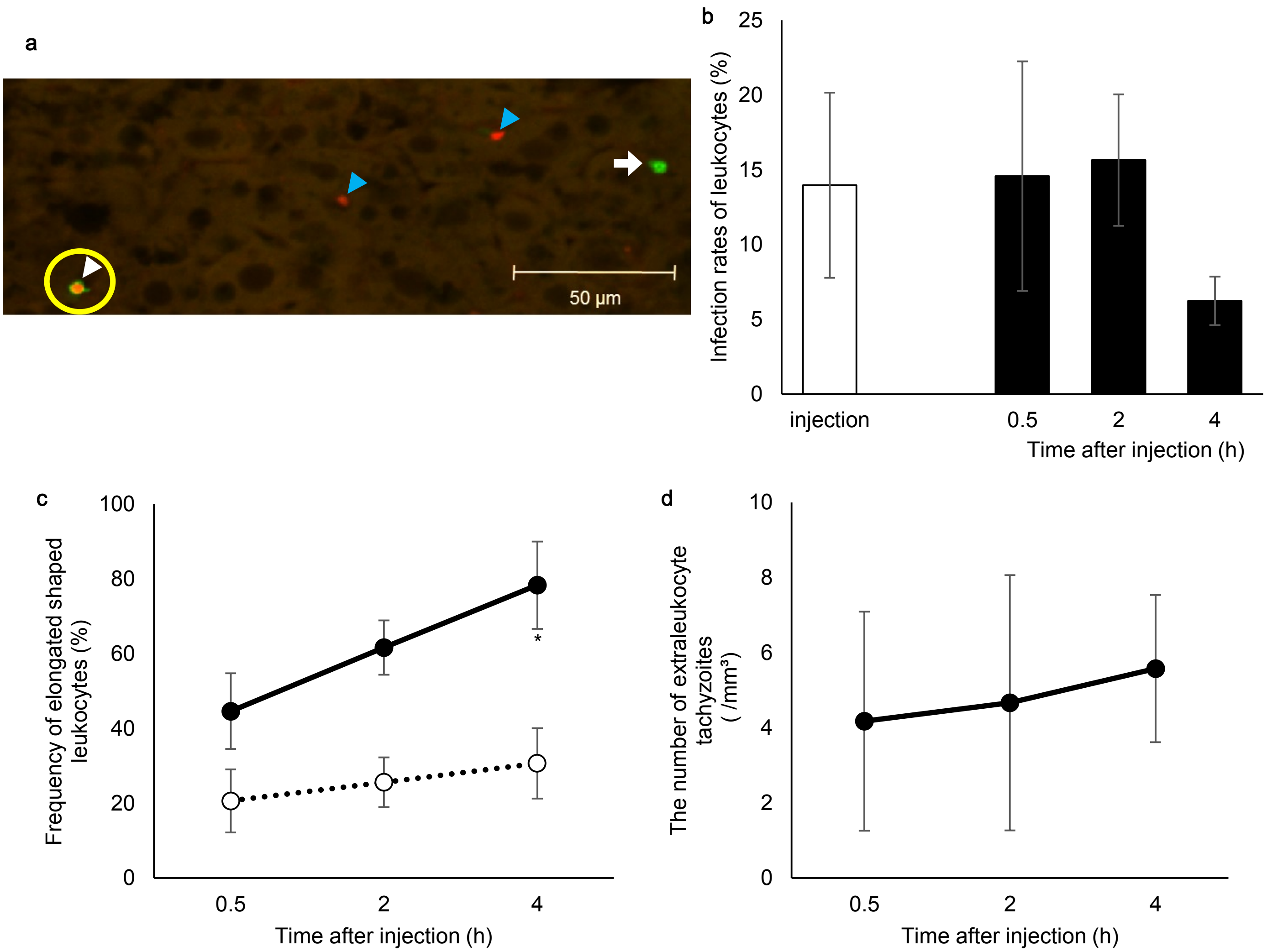

a
50 μm
b
Infection rates of leukocytes (%)
25
20
15
10
5
0
injection
0.5
2
4
Time after injection (h)
c
Frequency of elongated shaped leukocytes (%)
100
80
60
40
20
0
*
0.5
2
4
Time after injection (h)
d
The number of extraleukocyte tachyzoites (/mm³)
10
8
6
4
2
0
0.5
2
4
Time after injection (h)

Supplementary Figure 1: Tachyzoite-infected leukocytes and leukocyte-free tachyzoites in the liver

(a) Representative confocal scanning laser microscope image of the liver at 30 min post injection of leukocytes from a GFP mouse. The circle indicates a red fluorescent tachyzoite-infected leukocyte. The white arrow and arrowhead indicate non-infected and infected leukocytes, respectively. Blue arrowheads indicate extraleukocytic tachyzoites. (b) Frequency of tachyzoite-infected cells among total GFP-positive leukocytes at the point of injection (white) and in the liver (black). Results are presented as the mean ± S.E. Data were from three independent experiments and were analysed using one-way ANOVA, but no statistical differences were detected. (c) Frequency of elongated shaped leukocytes in the liver. Solid and dashed lines indicate the frequency of elongated shaped leukocytes among infected and non-infected GFP-positive leukocytes, respectively, in the liver. Results are presented as the mean ± S.E. Data were from three independent experiments and were analysed using two-way ANOVA. Scheffe F-test was performed as a post hoc analysis. *Statistical difference between infected and non-infected leukocytes ($p < 0.05$). (d) The number of extraleukocytic tachyzoites in the liver. Results are presented as the mean ± S.E. Data were from three independent experiments and were analysed using one-way ANOVA, but no statistical differences were detected.

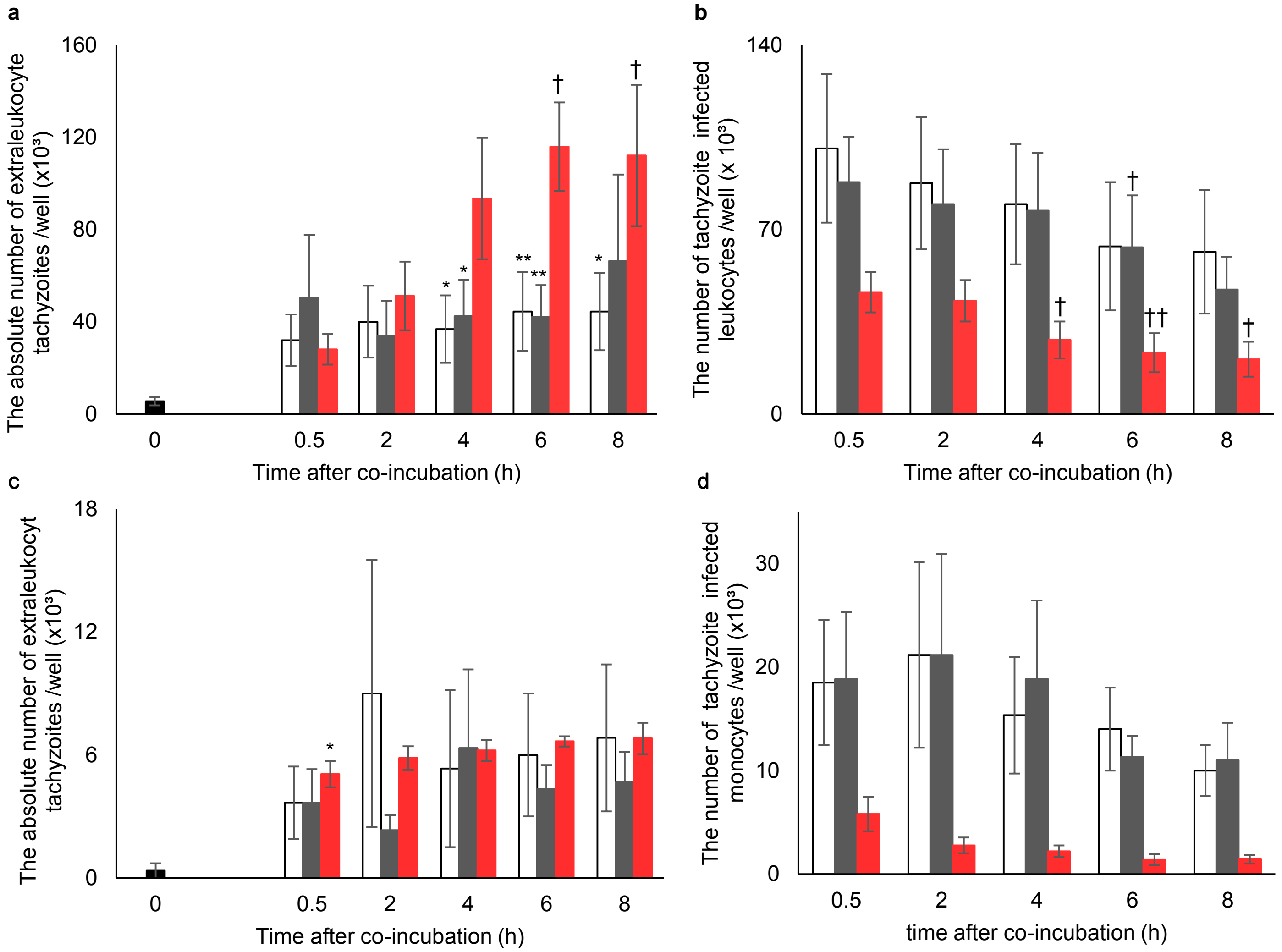
a
160
120
80
40
0
The absolute number of extraleukocyte tachyzoites /well (x10³)
0
0.5
2
4
6
8
Time after co-incubation (h)
b
140
70
0
The number of tachyzoite infected leukocytes /well (x 10³)
0.5
2
4
6
8
Time after co-incubation (h)
c
18
12
6
0
The absolute number of extraleukocyt tachyzoites /well (x10³)
0
0.5
2
4
6
8
Time after co-incubation (h)
d
30
20
10
0
The number of tachyzoite infected monocytes /well (x10³)
0.5
2
4
6
8
time after co-incubation (h)

Supplementary Figure 2: Absolute number of extracellular tachyzoites and tachyzoite-infected leukocytes/monocytes

(a and b) Absolute number of extraleukocytic tachyzoites and tachyzoite-infected leukocytes. The culture conditions are described in the legend for Fig. 2e. In the culture to analyse "attachment to lung endothelial cells (red)", non-attached cells were removed by washing 30 min after the co-culture was set up. Results are presented as the mean ± S.E. Data were from three independent experiments and were analysed using two-way ANOVA. Scheffe F-test was performed as a post hoc analysis. The statistical differences between leukocytes cultured alone (white), with endothelial cells separated by mesh (grey) or with endothelial cells directly (red) are indicated (* $p < 0.05$, ** $p < 0.01$). The statistical difference between each time point and the 30 min time point is also indicated († $p < 0.05$, †† $p < 0.01$). (c and d) The absolute number of extramonocytic tachyzoites and tachyzoite-infected monocytes. The culture conditions were the same as those described in the legend for Fig. 3c. Non-attached cells in the culture to analyse "attachment to lung endothelial cells (red)" were removed by washing 10 min after the co-culture was set up. Results are presented as the mean ± S.E. Data were from three independent experiments. (c) The statistical differences between 0 min (black: directly before co-incubation) and 30 min of co-incubation were analysed using the Student's t-test and the Holm method was used to adjust the P value when plural pairs were compared (* $p < 0.05$). (d) Statistical analysis was carried out as described for Supplementary Fig. 2b but no statistical difference was detected.

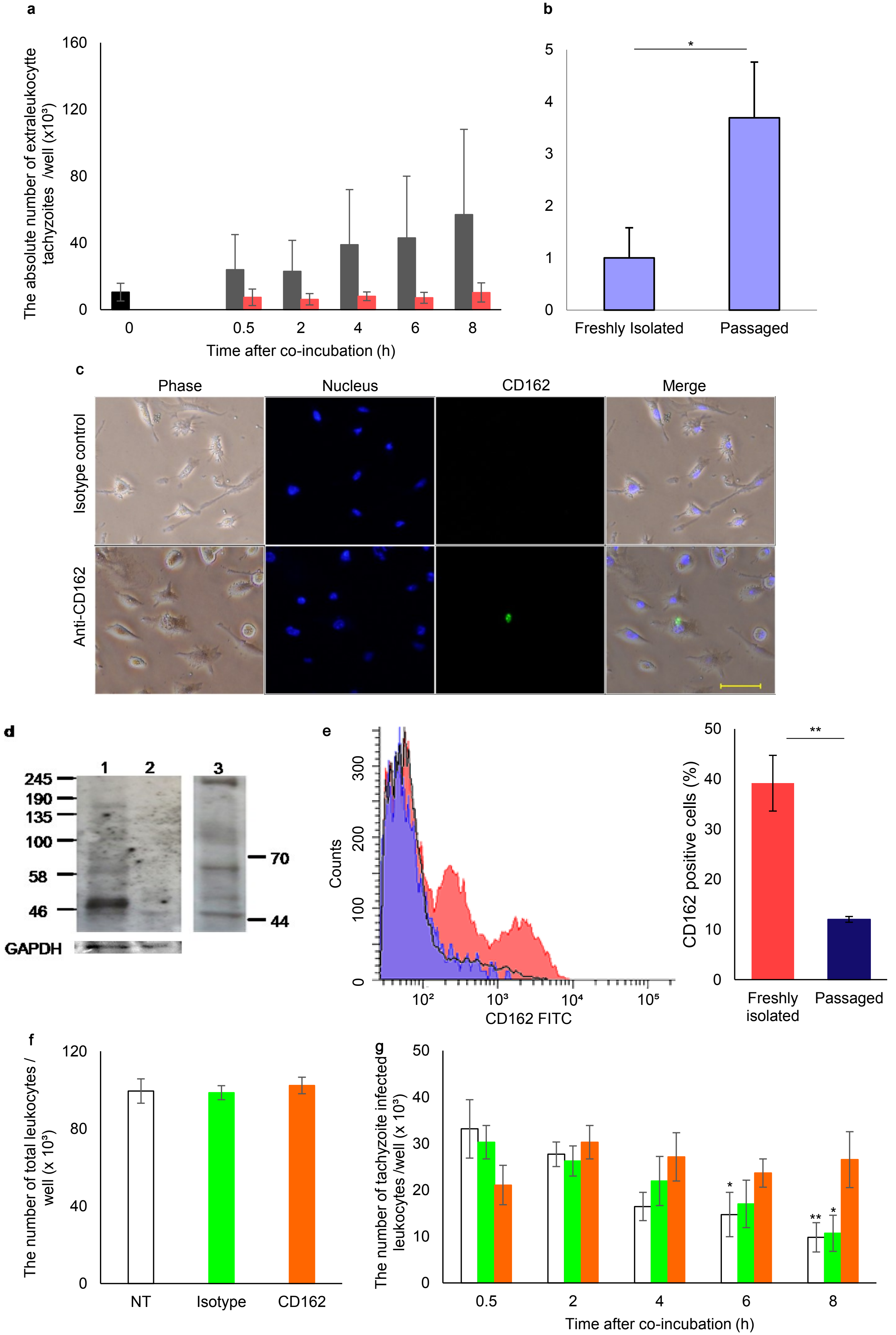
a
The absolute number of extraleukocytte tachyzoites /well (x10³)
160
120
80
40
0
0
0.5
2
4
6
8
Time after co-incubation (h)
b
*
5
4
3
2
1
0
Freshly Isolated
Passaged
c
Phase
Nucleus
CD162
Merge
Isotype control
Anti-CD162
d
245
190
135
100
58
46
1
2
3
70
44
GAPDH
e
Counts
300
200
100
0
10²
10³
10⁴
10⁵
CD162 FITC
CD162 positive cells (%)
50
40
30
20
10
0
**
Freshly isolated
Passaged
f
The number of total leukocytes / well (x 10³)
120
80
40
0
NT
Isotype
CD162
g
The number of tachyzoite infected leukocytes /well (x 10³)
50
40
30
20
10
0
*
**
*
0.5
2
4
6
8
Time after co-incubation (h)

Supplementary Figure 3: The effect of passaging of endothelial cells and CD162

(a) The absolute number of extraleukocytic tachyzoites was assessed. The culture conditions were the same as those described in the legend of Fig. 4c. Non-attached cells in the culture to analyse “attachment to passaged lung endothelial cells (red)” were removed by washing 30 min after the co-culture was set up. For the control, leukocytes were cultured with passaged endothelial cells separated by mesh (grey). The absolute number of extracellular tachyzoites at the start of co-culturing is shown as a black bar. Data were from three independent experiments and were analysed using two-way ANOVA. No statistical differences were detected between 0 min and the other time points. Results are presented as the mean ± S.E. (b) Relative icam-1 gene expression level in the freshly isolated and passaged endothelial cells. The icam-1 mRNA levels were quantified by real-time PCR using the gapdh gene as a standard. The expression level of the icam-1 gene in freshly isolated cells was standardized as 1. Results are presented as the mean ± S.E. Data were from three independent experiments. Statistical analysis was carried out using the Student’s t-test (* $p < 0.05$). (c and d) Lung endothelial cells were pre-treated with anti-CD162 antibody (orange), isotype control antibody (green) or PBS (white). (c) Immunostaining of freshly isolated lung endothelial cells without 0.5% formaldehyde treatment. Phase: images with transmitted light, Nucleus: staining with Hoechst, CD162: staining with isotype control (upper) or anti-CD162 antibody (lower), Merge: the combined image. Arrowhead shows CD162 and the scale bar indicates 50 μm. (d) Western blot analysis of CD162 protein expression in lung endothelial cells before (lane 1) and after passage (lane 2). Lane 3 shows leukocytes. (e) Left panel: flow cytometry histogram of lung endothelial cells. Lung endothelial cells were incubated with anti-CD162 FITC antibody (red: fleshly isolated, blue: passaged) or isotype control antibody (black solid line). Right panel: Percentage of CD162-positive cells was calculated in fleshly isolated lung endothelial cells (red) and passaged lung endothelial cells (blue), respectively. Results are presented as the mean ± S.E. Data were from three independent experiments. Statistical analysis was carried out using the Student’s t-test (** $p < 0.01$). (f) The total number of attached leukocytes is indicated. Statistical analysis was carried out using one-

way ANOVA. No statistical differences were detected. (g) The number of attached tachyzoite-infected leukocytes is indicated. Statistical analysis was carried out using one-way ANOVA between the 30-min time point and each of the other time points. Dunnett's test was performed as a post hoc analysis (* $p < 0.05$, ** $p < 0.01$). Results are presented as the mean ± S.E. Data were from three independent experiments.

Supplementary Table 1: Expression level of 88 adhesion-related genes.

Gene	expression level		SD	
	Fresh isolated	Passaged	Fresh isolated	Passaged
itgam	1.18E-03	2.69E-02	1.10E-03	1.67E-01
cdh2	1.31E-01	2.92E-01	4.16E-02	2.50E-01
cdh3	1.84E-03	4.14E-03	1.92E-03	2.87E-03
cntn1	6.20E-05	3.61E-04	3.44E-05	3.48E-04
sdc2	4.43E-02	1.34E-01	1.59E-02	1.11E-01
cd80	2.19E-03	6.92E-03	1.04E-03	4.68E-03
h2-ea	1.05E-03	2.75E-01		
h2-t10	1.97E-02	4.05E-02	2.17E-02	5.16E-02
h2-t23	4.91E-02	1.04E-01	3.53E-02	5.86E-02
mag	8.71E-05	5.55E-04	2.15E-05	1.32E-04
ncam1	1.70E-02	3.91E-02	1.06E-02	5.10E-02
sdc4	6.43E-01	1.40E+00	2.85E-01	9.09E-01
vcam1	1.16E+00	2.86E+00	2.86E-01	1.12E+00
nrxn2	9.01E-05	2.15E-04	5.20E-05	8.23E-05
jam2	1.17E-02	6.36E-02	7.41E-03	3.15E-02
nlgn1	5.40E-05	3.44E-04		2.11E-04
nrxn3	4.35E-05	3.84E-04	2.41E-05	2.89E-04
negr1	5.66E-04	1.60E-03	2.18E-04	1.36E-03
itgal	5.35E-04	1.50E-04	3.22E-04	2.11E-04
itgb2	4.69E-03	1.01E-03	1.51E-03	8.15E-04
l1cam	8.40E-04	3.10E-04	1.00E-03	4.17E-04
ocln	4.62E-03	2.03E-03	2.23E-03	2.42E-03
selplg	1.59E-03	1.28E-04	7.06E-04	9.29E-05
cdh1	5.81E-03	1.18E-03	6.48E-03	5.44E-04
cldn3	1.58E-03	3.64E-04	1.70E-03	2.10E-04
cldn4	5.06E-03	3.12E-04	5.41E-03	3.61E-04
ptprc	3.68E-03	3.66E-04	2.29E-03	4.05E-04
sell	5.13E-04	1.43E-04	1.47E-03	1.23E-04
siglec1	4.22E-03	2.81E-04	1.46E-03	3.80E-04
cldn7	1.29E-03	1.18E-04	1.60E-03	1.75E-04
cd86	3.39E-04	7.33E-05	1.58E-04	2.00E-05
itga8	1.55E-02	1.98E-02	9.52E-03	2.28E-02
h2-k1	1.70E-01	2.71E-01	1.68E-01	2.53E-01
cadm1	3.45E-03	5.54E-03	2.90E-03	4.84E-03
cd6	6.07E-05			
neo1	7.10E-03	6.41E-03	3.24E-03	4.06E-03
vcan	5.30E-02	3.70E-02	2.41E-02	3.31E-02
mpzl1	1.62E-01	1.70E-01	7.27E-02	1.86E-01
cd28	7.01E-04	4.45E-04	4.02E-04	2.27E-04
glycam1	4.76E-05	6.80E-05	1.50E-05	3.91E-05
h2-oa	5.63E-05			
h2-t24	9.93E-05	1.57E-04	4.43E-05	2.07E-04
itga6	3.90E-01	2.51E-01	2.44E-01	3.11E-01
cldn11	9.78E-04	1.75E-03	6.04E-04	1.57E-03
pdcd1		4.61E-05		
pecam1	7.44E-01	6.18E-01	4.63E-01	7.11E-01
ptprm	7.76E-02	8.22E-02	4.95E-02	9.44E-02
pvrl2	2.40E-01	2.26E-01	1.43E-01	2.13E-01
glg1	1.82E-01	1.85E-01	1.86E-01	2.17E-01
alcam	7.24E-02	1.14E-01	2.69E-02	9.82E-02
cdh5	1.20E+00	1.04E+00	1.12E+00	1.25E+00

h2-aa	5.67E-05	7.86E-05	4.64E-05	
h2-d1	1.46E-01	1.86E-01	7.50E-02	1.39E-01
h2-eb1	1.37E-04	2.15E-04	1.61E-04	2.09E-04
h2-dmb2	7.22E-05			
h2-ob				
h2-t22	2.88E-02	5.73E-02	2.57E-02	6.17E-02
icam2	1.35E-01	1.49E-01	3.56E-02	1.53E-01
itga4	6.27E-03	8.52E-03	3.02E-03	7.25E-03
itgb1	1.04E+00	1.39E+00	7.46E-01	1.34E+00
ncam2	1.22E-04	8.37E-05		6.70E-05
ptprf	4.52E-02	5.42E-02	2.29E-02	3.19E-02
selp	9.35E-01	1.40E+00	7.00E-01	1.61E+00
sdc1	2.84E-02	2.19E-02	8.94E-03	1.62E-02
sdc3	1.34E-01	1.25E-01	7.53E-02	1.02E-01
cd40	1.33E-02	1.92E-02	1.35E-02	2.48E-02
cd2		3.28E-04		
cd4	4.50E+02			
itgb7	9.01E-05	9.88E-05	6.05E-05	4.56E-05
cldn1	2.81E-03	4.38E-03	1.86E-03	4.74E-03
cntnap1	8.34E-04	1.00E-03	5.39E-04	9.92E-04
icos	1.72E-04	1.70E-04	8.06E-05	1.25E-04
cldn8		7.18E-05		
pvrl3	4.16E-02	5.20E-02	1.72E-02	3.97E-02
cldn15	5.78E-04	9.60E-04	2.54E-04	2.47E-04
cd274	3.50E-03	5.07E-03	1.01E-03	5.19E-03
jam3	1.79E-02	2.62E-02	1.31E-02	2.65E-02
esam1	6.35E-02	7.36E-02	2.42E-02	4.72E-02
cldn16		2.28E+03		
cd34	8.32E-02	5.89E-02	2.17E-02	3.83E-02
itga9	1.91E-02	2.28E-02	1.23E-02	2.02E-02
cd276	6.95E-02	6.58E-02	3.11E-02	5.26E-02
f11r	3.77E-01	4.13E-01	1.89E-01	3.73E-01
nlgn3	1.28E-04	1.20E-04	8.53E-05	7.52E-05
nrcam	2.35E-04	1.95E-04	2.54E-04	2.29E-04
itgb8	1.05E-03	1.58E-03	7.43E-04	1.42E-03
h2-ab1	1.26E-04	1.26E-04	1.41E-04	7.37E-05
h2-q6	2.78E-01	4.91E-01	3.12E-01	5.13E-01

Expression level of each gene was normalized based on data from eight house-keeping genes. Cut-off value of Ct was 35.

Supplementary Video 1: Egression of *T. gondii* tachyzoites from leukocytes .

A single tachyzoite egressed from an infected leukocyte attached to lung endothelial cells.

Supplementary Video 2: Rapid egression of tachyzoites from monocytes.

Tachyzoites egressed from an infected monocyte attached to lung endothelial cells within 30 min.
